# Disseminated Corynebacterium jeikeium Infection in Cancer Patients

**DOI:** 10.7759/cureus.8764

**Published:** 2020-06-22

**Authors:** Shylah M Moore Pardo, Raj H Patel, Asha Ramsakal, John Greene

**Affiliations:** 1 Internal Medicine, University of South Florida, Tampa, USA; 2 Internal Medicine, Moffitt Cancer Center, Tampa, USA

**Keywords:** corynebacterium jeikeium, septic emboli, acute myelogenous leukemia, corynebacterium, multidrug-resistant pathogen

## Abstract

*Corynebacterium jeikeium* is a multidrug-resistant gram-positive bacterium of the human skin flora and one of the most clinically important nondiphtherial corynebacteria in the acute care setting. *C. jeikeium* can cause different forms of infections, especially in immunocompromised patients with underlying risk factors and comorbidities. *C. jeikeium* was initially described in 1976 as a highly resistant coryneform bacteria causing severe sepsis in patients with hematologic malignancies and profound neutropenia. *C. jeikeium* infection has also been reported in the setting of endocarditis, septicemia, meningitis, pneumonia, and soft tissue infections. Management of disseminated *C. jeikeium* infection in immunocompromised cancer patients can be challenging due to its high virulence and rapid skin colonization. We present two cases of disseminated *C. jeikeium* infection in patients with acute myelogenous leukemia (AML) and underlying comorbidities. Both patients presented with neutropenic fever resistant to initial standard empiric antibiotic therapy.

## Introduction

Nondiphtherial corynebacteria are gram-positive rods that are increasingly recognized as etiologic pathogens for different human infections. *Corynebacterium jeikeium* is a highly virulent, multidrug-resistant pathogen that should not be considered a contaminant, especially amongst immunocompromised patients with risk factors. Risk factors for infection include immunocompromised state, hematologic malignancy, neutropenia, prior antibiotic exposure, and presence of an intravascular catheter [1]. Prolonged hospital stay, treatment with broad-spectrum antibiotics, and impaired skin integrity are risk factors for infection with *C. jeikeium* [2]. *C. jeikeium *colonizes the skin of hospitalized patients, especially those treated with multiple antibiotics, and can also be isolated from the hospital environment. In the acute care setting, *C. jeikeium *is the most clinically important pathogen of the nondiphtherial corynebacterial class. Previous reports have indicated cutaneous manifestations from *C. jeikeium* in the setting of sepsis [3]. Pulmonary infiltrates in patients with acute myelogenous leukemia (AML) have also been reported to occur with *C. jeikeium* bacteremia [4]. Septic emboli generally occur as a result of disseminated infection and bacteremia, whereas pneumonia does not always accompany bacteremia and is an independent pulmonary process. These differences can be distinguished through imaging and clinical signs. In the case of both complications occurring simultaneously, high morbidity is likely and patients generally require intensive care. *C. jeikeium* is resistant to antibiotic therapy with penicillins, cephalosporins, and aminoglycosides. However, it remains susceptible to vancomycin, which is the recommended primary treatment regimen [5,6]. We report two patients undergoing chemotherapy for AML who developed disseminated *C. jeikeium *infection along with prolonged neutropenia. The first patient presented with septic emboli to the lungs and skin without evidence of endocarditis by transthoracic echocardiogram. The second patient presented with miliary nodular pneumonia with skin lesions.

## Case presentation

Case 1

The first patient is a 65-year-old male diagnosed with AML three months prior to admission. He initially underwent induction chemotherapy consisting of daunorubicin and cytarabine (Vyxeos). He was admitted and received salvage chemotherapy consisting of cladribine, cytarabine, granulocyte colony-stimulating factor (G-CSF), and mitoxantrone (CLAG-M) for treatment of refractory AML. Upon physical examination, the elderly patient appeared frail, acutely ill, and toxic. Blood pressure (BP) taken upon admission was 114/74 mmHg, pulse rate (PR) 98 beats per minute, temperature 102.6°F, and respiratory rate (RR) 24 breaths per minute. The patient had dry oral mucosa without evidence of oral lesions, exudates, or pharyngeal erythema. Tachycardia on auscultation without murmur was appreciated on the cardiac exam. The lungs were clear bilaterally and respirations were nonlabored. The abdominal exam was benign.

**Figure 1:**
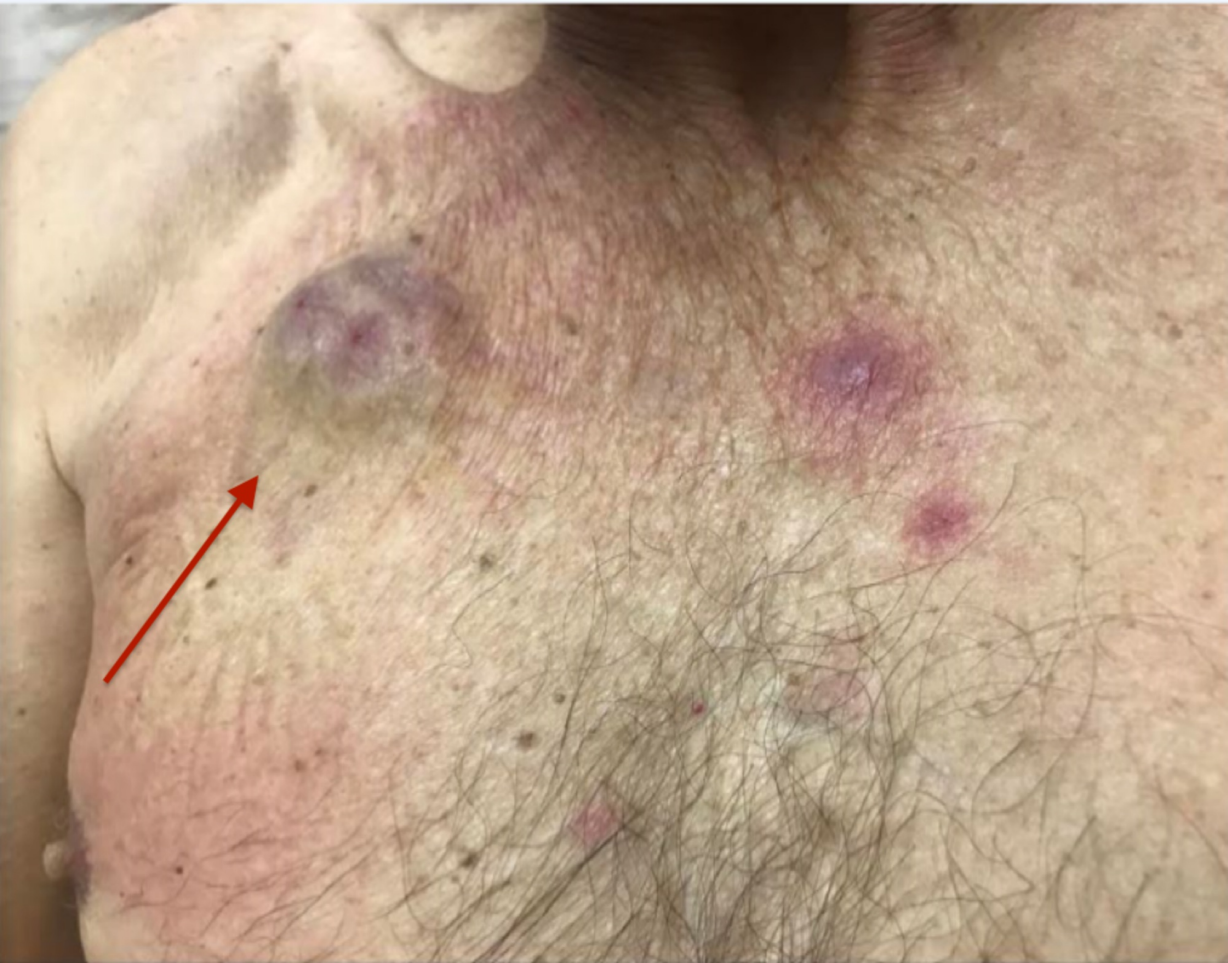
Port site erythema on the right chest

A baseline CT scan of the thorax done on admission demonstrated bibasilar atelectasis and scarring but no mass, consolidations, or suspicious pulmonary nodules were detected. A baseline CT scan of the sinuses had no evidence of sinusitis. Methicillin-resistant *Staphylococcus aureus (*MRSA) screening was negative. The white blood cell count was 0.21 thousand per cubic millimeter (normal reference range: 4.00-10.90) with an absolute neutrophil count of 0 cells per microliter (normal reference range: 1.5-8.0) indicative of profound neutropenia. Procalcitonin levels were slightly elevated at 1.29 nanograms per milliliter (normal reference range: 0.02-0.49). The respiratory viral panel was negative for adenovirus, coronavirus, human metapneumovirus, human rhinovirus/enterovirus, influenza, parainfluenza, and respiratory syncytial virus. Subsequently, oral acyclovir, ciprofloxacin, and voriconazole prophylaxis were begun on the first day of neutropenia when the absolute neutrophil count (ANC) reached 260 cells/mm^3^. He was also found to have mild right chest port erythema without pustular discharge, which resolved after a seven-day course of oral doxycycline was added empirically (Figure 1). On day 11 of neutropenia, the patient developed sepsis along with a fever and a new rash. Blood and urine cultures were collected. Oral ciprofloxacin was switched to intravenous piperacillin/tazobactam. The patient continued to have a fever despite the optimization of antibiotics. He also developed mild shortness of breath, difficulty breathing, and dry cough.

**Figure 2:**
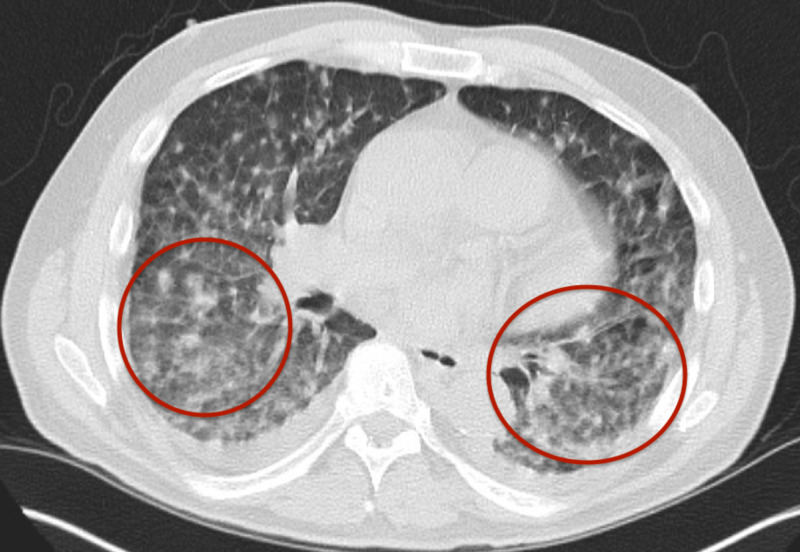
CT of the chest showing multiple bilateral septic emboli

Blood cultures four days after collection grew gram-positive rods and intravenous vancomycin was added empirically. A CT scan of the chest was repeated due to worsening hypoxia and revealed findings consistent with multiple septic emboli (Figure 2). A plain radiograph of the chest was repeated upon onset of neutropenic fever and revealed new right infrahilar opacity. There was no evidence of vegetation or endocarditis on echocardiogram. He was placed on 4-6 liters of supplemental oxygen. After 21 days of profound neutropenia, blood and tissue cultures grew *C. jeikeium*. The presence of nodular skin lesions that were consistent with septic emboli was concerning for disseminated disease. He had no Janeway lesions or Osler’s nodes.

**Figure 3:**
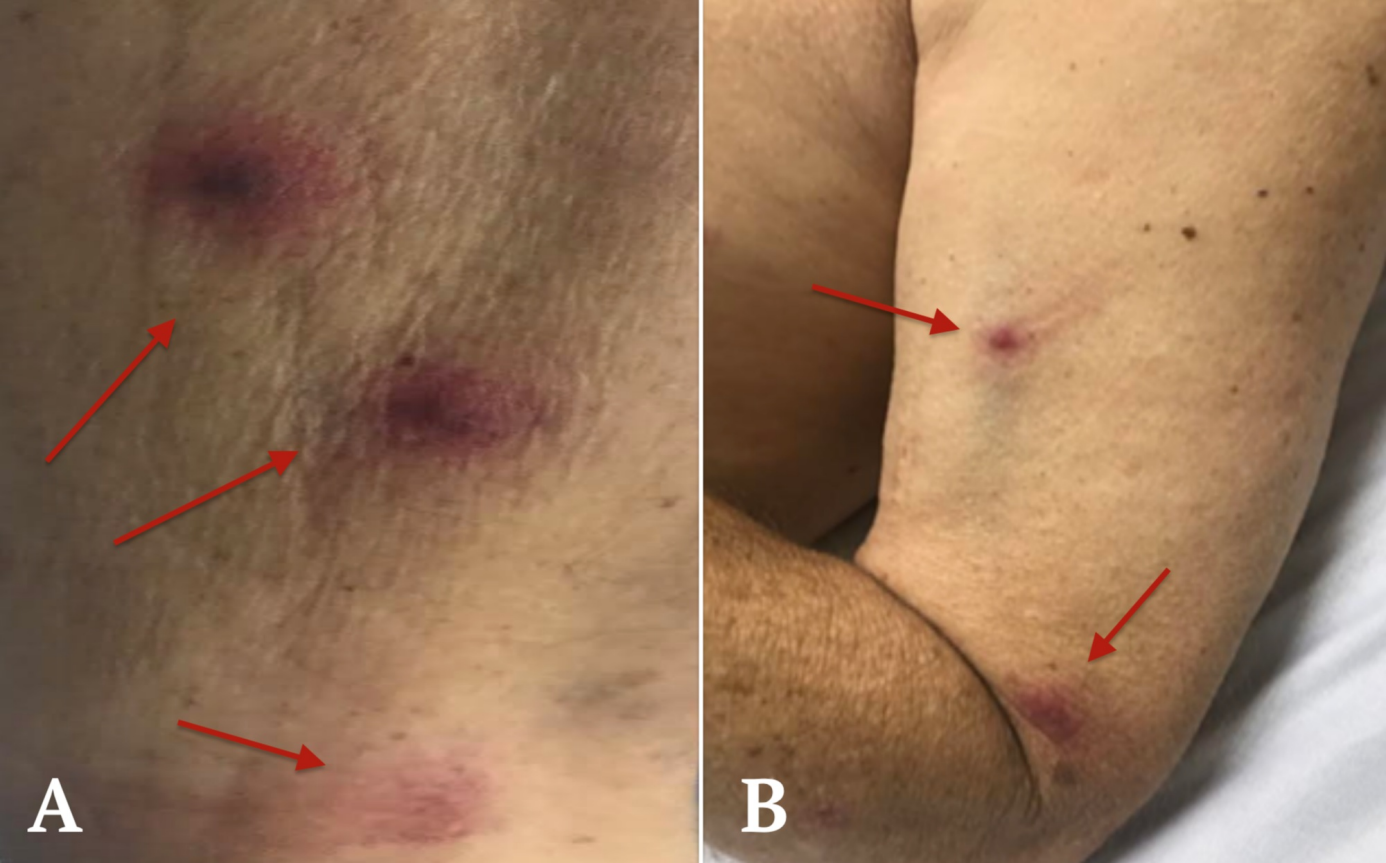
Skin lesions on the right and left arms (A) Presence of skin lesions on the right arm. (B) Presence of skin lesions on the left arm.

Skin exam demonstrated multiple nonblanching, nonpruritic, minimal tender, red-violaceous papulonodular lesions without crusting on the anterior chest, and both upper extremities (Figure 3). A subungual nail hemorrhage was observed on the fourth digit of the left hand. Skin biopsy showed positive results for the presence of *C. jeikeium* and diphtheroids. 

Further susceptibilities were requested and sent out to a reference lab. This demonstrated multidrug resistance to clindamycin, penicillin, and gentamicin but susceptibility to vancomycin with a minimum inhibitory concentration (MIC) of 0.5 micrograms per milliliter. The port was subsequently removed, and repeat blood cultures were taken. After several weeks of continued therapy, his neutropenia and fever resolved. Other than easy fatigability, he was able to ambulate normally and felt well enough to resume normal activities. He was discharged with six weeks of intravenous vancomycin which cured his infection. His respiratory symptoms and diffuse rash eventually resolved. A CT scan of his chest was repeated at the end of the antibiotic course which was negative. He was then referred for bone marrow transplant evaluation a few months after completing therapy.

Case 2

The second patient was a 68-year-old male undergoing chemotherapy with Vyxeos induction for treatment of AML. Upon physical examination, the patient’s BP was 136/88 mmHg, PR 74 beats per minute, temperature 99.8°F, and RR 22 breaths per minute. The patient had no pharyngeal erythema, lungs were clear to auscultation, and respirations were nonlabored. The abdominal exam was also normal. The patient had a history of extensive travel, had a transient ischemic attack (TIA) two years prior to admission, and latent tuberculosis. A baseline CT of the chest and sinuses was negative. The patient was diagnosed with neutropenic fever. Empiric antibiotics were initiated. One month after profound neutropenia, blood cultures were positive for *C. jeikeium* infection. Further susceptibilities were requested and sent out to a reference lab. This demonstrated resistance to penicillin, gentamicin, clindamycin, and susceptibility to daptomycin with an MIC of 0.5 micrograms per milliliter.

**Figure 4:**
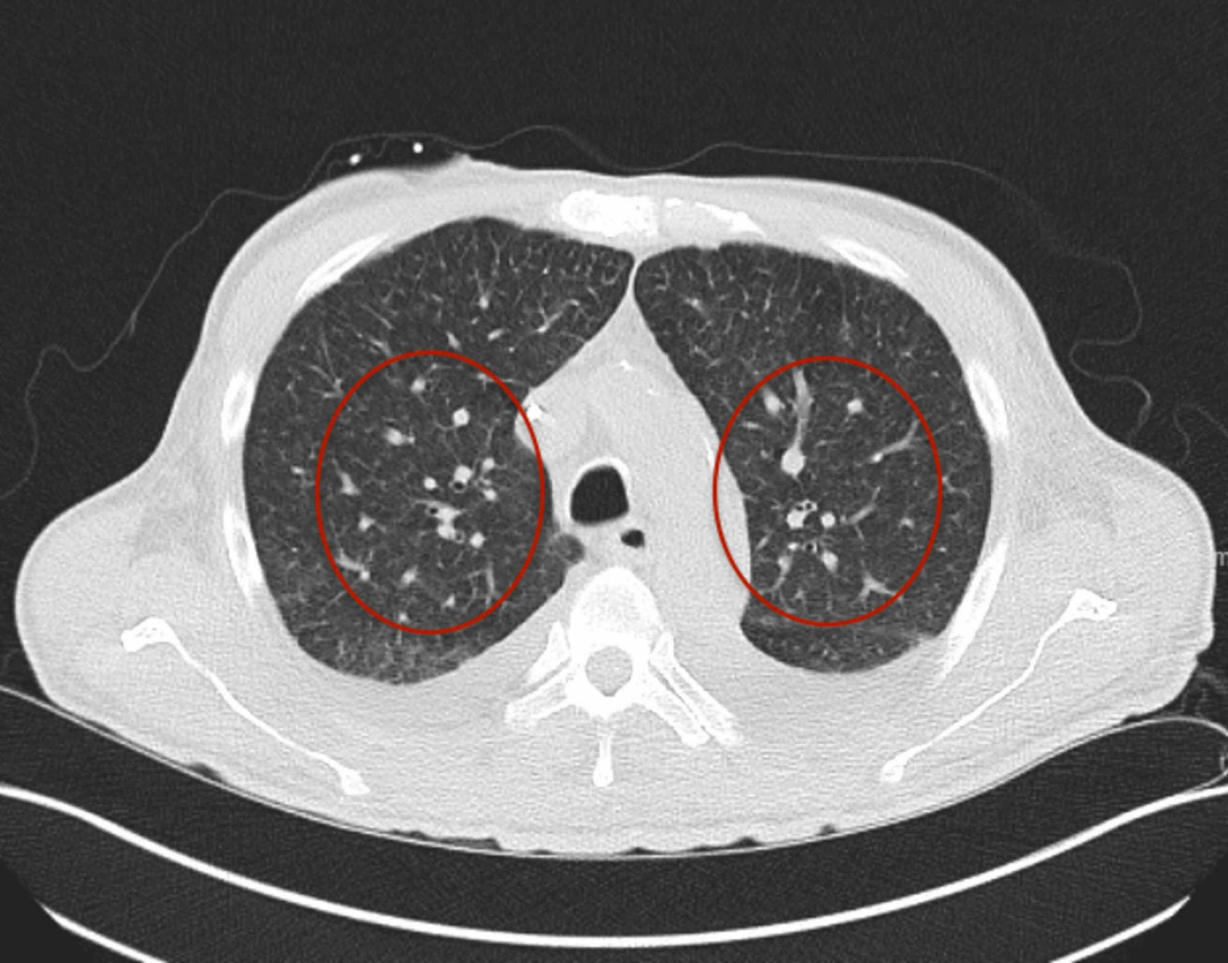
CT of the chest showing small miliary nodules in the lungs consistent with septic emboli

The patient also had a rash and skin lesions which were consistent with septic emboli from *C. jeikeium* infection. The presence of skin lesions was concerning for the possibility of dissemination of infection. CT scan of the chest from two days after positive blood culture showed the presence of bilateral small miliary nodules consistent with septic emboli throughout the lungs (Figure 4). A repeat CT scan done 13 days later showed larger pulmonary nodules, and a follow-up CT scan was done 13 days after this showered worsening pulmonary nodules and increased pleural effusion. He had no respiratory symptoms, and the respiratory viral panel was negative. He remained neutropenic for 21 days after the positive blood culture.

Skin examination revealed small bilateral buttock eschars with no surrounding erythema, and presence of dark red circular skin lesions on the left upper extremities, chest, trunk, and legs. There were no lesions on his palms or soles. *C. jeikeium* bacteremia lasted for over a month despite aggressive antimicrobial therapy with intravenous vancomycin, levofloxacin, isavuconazonium sulfate, isavuconazole, and meropenem. Port removal was discussed but denied by the patient. Despite continued antimicrobial therapy, the patient passed away in hospice care due to complications with refractory AML and neutropenia prior to completing the full course of treatment.

## Discussion

*C. jeikeium* is a highly virulent, multidrug-resistant organism that should never be considered a contaminant in immunocompromised patients with risk factors. *C. jeikeium *is one of the most resistant *Corynebacterium *spp. with respect to antimicrobial agents. The majority of *C. jeikeium* clinical isolates are resistant to antibiotics such as beta-lactams and macrolides, and often remain susceptible only to glycopeptides [7]. In both of our cases, *C. jeikeium* was resistant to all antimicrobial agents except for vancomycin. One-third of patients with hematological disease and *C. jeikeium *infection present with a high incidence of pulmonary lesions. In our case, given that the neutropenic patient in case 1 had a device-associated infection, empiric antibiotic therapy with vancomycin was the optimal drug of choice as per the Infectious Diseases Society of America (IDSA) guidelines [4,8]. A prolonged course of antibiotic therapy of six weeks should be given if dissemination is suspected.

Given the small time interval between our two cases, matching demographics and risk factors, and evidence that patient-to-patient transmission in the hospital is a possible risk factor for *C. jeikeium* infection, hospital infection control was consulted but determined that there was no relation between the two cases. Both of our patients were immunosuppressed and had numerous risk factors predisposing them to this infection, thus causing them to develop prolonged neutropenia which was resistant to antibiotic therapy. Predisposing risk factors for *C. jeikeium* infection can also include immunocompromised states, hematologic malignancy, prolonged neutropenia, and AIDS. The presence of indwelling medical devices, such as intravascular catheters, peritoneal dialysis catheters, prosthetic valves, and cerebrospinal fluid (CSF) shunts, has also been described. Prolonged hospital stay, treatment with broad-spectrum antibiotics, and impaired skin integrity are also well-described risk factors for the development of infection with *C. jeikeium *[2].

Neutropenia is an important predisposing factor for infection in cancer patients. Susceptibility to bacterial and fungal infections increases significantly in neutropenic patients with an ANC of less than 500 cells/mm^3^ [9]. Multidrug-resistant gram-negative bacilli, such as Enterobacteriaceae and *Pseudomonas, *and gram-positive cocci, such as staphylococci and streptococci, have been described in the setting of neutropenia [2]. *Staphylococcus aureus* infection has also been found to cause septic pulmonary emboli in neutropenic patients [10]. Neutropenic patients with hematologic malignancies are also at high risk for life-threatening cutaneous fungal infections from organisms such as *Candida*, *Fusarium*, and *Rhizopus*. These infections present a high risk of mortality due to resistance to antifungal therapy and the rapid dissemination of infection. Immediate initiation of empiric antibiotic therapy is thus a key factor in reducing mortality in febrile neutropenic patients with disseminated infection [11]. Like *C. jeikeium*, clinical manifestations from these organisms include skin lesions from hematogenous dissemination [12].

Septic emboli can cause both skin and pulmonary lesions in neutropenic patients. The presence of intravascular devices has also been described as a major risk factor for infection with *C. jeikeium* [8]. Skin lesions can also be an important marker of disseminated life-threatening infection, and early detection can play a key role in prompt management [13]. In case 1, skin biopsy demonstrated positive results for the presence of *C. jeikeium* and diphtheroids. This proved that the skin lesions were a direct result of the dissemination of infection. Skin examinations, biopsies, and awareness of various clinical features of skin lesions are thus important for expedited diagnosis and therapy for patients infected with multidrug-resistant organisms [14]. Interestingly, both cases had lesions that spared the palms and soles. These studies in conjunction with blood culture reports are vital for diagnosis as septic emboli may appear before symptoms develop or positive blood cultures. 

## Conclusions

Our two cases highlight the challenges associated with the management of *C. jeikeium* in the cancer setting. These cases also emphasize the importance of maintaining a high index of clinical suspicion for *C. jeikeium* infection in immunocompromised patients resistant to antimicrobial therapy who present with skin and pulmonary lesions, neutropenia, and hematologic malignancies. Given the rapid skin colonization and high virulence of *C. jeikeium*, the management of clinical symptoms can be particularly challenging in immunocompromised cancer patients given the limited treatment options. Catheter-related infection can further complicate management and restrict treatment options as seen in our two cases. Prompt intervention with empirical intravenous vancomycin therapy along with a prolonged course of antibiotic therapy is recommended in patients with septic emboli, catheter-associated infection, and disseminated disease.
